# Integrated analysis of chromosome copy number variation and gene expression in cervical carcinoma

**DOI:** 10.18632/oncotarget.22403

**Published:** 2017-11-11

**Authors:** Deng Yan, Song Yi, Wang Chi Chiu, Liu Gui Qin, Wong Hoi Kin, Chung Tony Kwok Hung, Han Linxiao, Choy Kwong Wai, Sui Yi, Yang Tao, Tang Tao

**Affiliations:** ^1^ Department of Obstetrics & Gynaecology, The Chinese University of Hong Kong, Hong Kong, China; ^2^ CUHK Shenzhen Research Institute, Shenzhen, China; ^3^ Shenzhen Laboratory of Ophthalmology, Shenzhen Eye Hospital, Affiliated Shenzhen Eye Hospital of Shenzhen University, Shenzhen, China; ^4^ Dongguan Third People’s Hospital, Dongguan, China; ^5^ Department of Nutrition, The First Affiliated Hospital of Sun Yat-sen University, Guangzhou, China; ^6^ Center for Medical Research and Innovation, Shanghai Pudong Hospital, Fudan University Pudong Medical Center, Shanghai, China

**Keywords:** cervical cancer, chromosome copy number variation, gene expression, cluster analysis, cell cycle pathways

## Abstract

**Objective:**

This study was conducted to explore chromosomal copy number variations (CNV) and transcript expression and to examine pathways in cervical pathogenesis using genome-wide high resolution microarrays.

**Methods:**

Genome-wide chromosomal CNVs were investigated in 6 cervical cancer cell lines by Human Genome CGH Microarray Kit (4x44K). Gene expression profiles in cervical cancer cell lines, primary cervical carcinoma and normal cervical epithelium tissues were also studied using the Whole Human Genome Microarray Kit (4x44K).

**Results:**

Fifty common chromosomal CNVs were identified in the cervical cancer cell lines. Correlation analysis revealed that gene up-regulation or down-regulation is significantly correlated with genomic amplification (*P*=0.009) or deletion (*P*=0.006) events. Expression profiles were identified through cluster analysis. Gene annotation analysis pinpointed cell cycle pathways was significantly (*P*=1.15E-08) affected in cervical cancer. Common CNVs were associated with cervical cancer.

**Conclusion:**

Chromosomal CNVs may contribute to their transcript expression in cervical cancer.

## INTRODUCTION

Although the most important etiological agent in cervical cancer is human papillomavirus (HPV) infection, only a small proportion of infected women developed cervical cancer. HPV infection alone is insufficient to induce malignant changes. Copy number variation (CNV) is a very common phenomenon in cervical cancer and may be important in its pathogenesis [1]. Comparative genomic hybridization (CGH) studies for cervical cancer progression have shown that chromosome 3q gain was associated with the transition from pre-invasive to invasive cervical carcinoma. Subsequently, array-based CGH (aCGH), where arrays of genomic sequences replaced metaphase chromosomes as hybridization targets, was established. More detailed and precise genomic variations had been found in cervical cancer by using aGCH [2]. Lando et al. reported several potential driver genes for cervical carcinogenesis aCGH [3]. However, a recent study reported a limited correlation between chromosomal CNV and gene expression by single nucleotide polymorphisms array platform [4].

In this study, a high resolution Human Genome CGH Microarray Kit was used to detect genome-wide chromosomal CNV in 6 cervical cancer cell lines. We also performed gene expression studies in the cervical cancer cell lines, cervical carcinoma tissues and normal cervical epithelia by using Whole Human Genome Microarray Kit. Using appropriate bioinformatics software, we identified several chromosomal CNV regions and aberrantly expressed genes in cervical cancer. Statistical analysis and gene annotation analysis were also performed for the array data.

## RESULTS

### Fifty common chromosomal CNVs were identified in cervical cancer cell lines

Using aCGH at a genome-wide resolution of 250 kb, a total of 50 common chromosomal CNV regions were identified, ranging from 0.5 Mb to 80 Mb. Of these, 21 common amplification regions (including 13 significant amplification regions) and 29 deletion regions (including 2 significant deletion regions) were identified (Table 1).

**Table 1:** Physical location of the chromosome CNV regions identified by array CGH in cervical cancer cell lines

Region	Region length	Cytoband location	Event	Genes	Frequency %	P-value	Reference
chr1:10001-3752828	3,742,828	p36.33 - p36.32	Gain	70	83.33333333	0.003	Gopeshwar Narayan, et al., 2007; Connie P. Matthews, et al., 2000; Y.W. CHOI, et al., 2007
chr2:75161025-85137285	9,976,261	p12 - p11.2	Loss	17	83.33333333	0.006	Y.W. CHOI^*^, et al., 2007
chr2:137,395,646-170,227,851	32,832,206	q21.3 - q31.1	Loss	84	100	>0.05	F.Y. Huang et al.2005; Y.W. CHOI, et al., 2007
chr2:178,374,598-197,908,813	19,534,216	q31.2 - q33.1	Loss	67	83.33333333	>0.05	F.Y. Huang et al.2005; Y.W. CHOI, et al., 2007
chr2:209,391,516-216,053,786	6,662,271	q34 - q35	Loss	15	83.33333333	>0.05	Lockwood WW, et al., 2007; F.Y. Huang et al.2005; G Ng, et al., 2007
chr3:60,001-8,582,632	8,522,632	p26.3 - p25.3	Loss	15	83.33333333	>0.05	F.Y. Huang et al.2005; Connie P. Matthews, et al., 2000; Y.W. CHOI, et al., 2007
chr3:16,297,340-37,460,259	21,162,920	p25.1 - p22.2	Loss	60	83.33333333	>0.05	Lockwood WW, et al., 2007; F.Y. Huang et al.2005; Connie P. Matthews, et al., 2000
chr3:58,690,297-90,181,487	31,491,191	p14.2 - p11.1	Loss	52	83.33333333	0.028	Lockwood WW, et al., 2007; F.Y. Huang et al.2005; Connie P. Matthews, et al., 2000; Y.W. CHOI, et al., 2007; G Ng, et al., 2007
chr3:93,605,515-101,219,924	7,614,410	q11.2 - q12.3	Loss	38	83.33333333	>0.05	N/A
chr4:10,479,679-39,591,168	29,111,490	p16.1 - p14	Loss	62	83.33333333	>0.05	Lockwood WW, et al., 2007; F.Y. Huang et al.2005; Y.W. CHOI, et al., 2007; G Ng, et al., 2007
chr4:41,596,003-44,365,208	2,769,206	p13	Loss	10	83.33333333	>0.05	Lockwood WW, et al., 2007; F.Y. Huang et al.2005; G Ng, et al., 2007
chr4:58,367,789-139,871,504	81,503,716	q12 - q31.1	Loss	300	83.33333333	>0.05	Lockwood WW, et al., 2007; G Ng, et al., 2007
chr4:141,329,535-182,773,601	41,444,067	q31.1 - q34.3	Loss	127	83.33333333	>0.05	Lockwood WW, et al., 2007; Y.W. CHOI, et al., 2007; G Ng, et al., 2007
chr5:49,690,172-58,526,175	8,836,004	q11.1 - q11.2	Loss	35	83.33333333	>0.05	Connie P. Matthews, et al., 2000
chr6:44,372,753-58,614,061	14,241,309	p21.1 - p11.2	Loss	69	83.33333333	>0.05	Connie P. Matthews, et al., 2000; Y.W. CHOI, et al., 2007
chr6:61,982,931-73,725,450	11,742,520	q11.1 - q13	Loss	16	83.33333333	>0.05	Connie P. Matthews, et al., 2000
chr6:75,067,155-105,246,238	30,179,084	q13 - q21	Loss	83	83.33333333	>0.05	Connie P. Matthews, et al., 2000
chr6:112,398,800-148,256,165	35,857,366	q21 - q24.3	Loss	143	83.33333333	>0.05	Connie P. Matthews, et al., 2000; Y.W. CHOI, et al., 2007
chr7:76,075,269-97,170,202	21,094,934	q11.23 - q21.3	Loss	77	83.33333333	>0.05	N/A
chr7:105,159,121-110,522,522	5,363,402	q22.3 - q31.1	Loss	24	83.33333333	>0.05	N/A
chr8:12,830,587-20,081,624	7,251,038	p22 - p21.3	Loss	27	83.33333333	>0.05	Lockwood WW, et al., 2007; G Ng, et al., 2007
chr8:75,336,800-85,510,468	10,173,669	q21.11 - q21.2	Loss	27	83.33333333	>0.05	N/A
chr8:142,141,881-146,304,022	4,162,142	q24.3	Gain	92	83.33333333	0	Gopeshwar Narayan, et al., 2007; F.Y. Huang et al.2005; Connie P. Matthews, et al., 2000; Y.W. CHOI, et al., 2007; G Ng, et al., 2007
chr9:128,223,213-139,309,447	11,086,235	q33.3 - q34.3	Gain	211	83.33333333	0.017	Lockwood WW, et al., 2007; Y.W. CHOI, et al., 2007
chr11:60,001-3,696,670	3,636,670	p15.5 - p15.4	Gain	85	83.33333333	0	Y.W. CHOI, et al., 2007; G Ng, et al., 2007
chr11:20,658,997-31,795,373	11,136,377	p15.1 - p13	Loss	27	83.33333333	>0.05	F.Y. Huang et al.2005
chr11:65,627,563-67,839,841	2,212,279	q13.1 - q13.2	Gain	77	83.33333333	0.003	Y.W. CHOI, et al., 2007
chr12:38,766,104-42,827,028	4,060,925	q12	Loss	12	83.33333333	>0.05	N/A
chr13:45,697,630-55,761,181	10,063,552	q14.12 - q21.1	Loss	56	83.33333333	>0.05	Lockwood WW, et al., 2007; G Ng, et al., 2007
chr14:23,251,874-24,898,017	1,646,144	q11.2- q12	Gain	64	83.33333333	0.001	N/A
chr16:60,001-3,153,334	3,093,334	p13.3	Gain	137	83.33333333	0.048	Y.W. CHOI, et al., 2007
chr16:3,970,244-5,071,063	1,100,820	p13.3	Gain	23	83.33333333	0.048	Y.W. CHOI, et al., 2007
chr16:28,276,920-31,195,342	2,918,423	p11.2	Gain	100	83.33333333	0.048	Y.W. CHOI, et al., 2007
chr16:66,279,668-70,782,910	4,503,243	q21 - q22.1	Gain	103	83.33333333	0.021	Lockwood WW, et al., 2007; Connie P. Matthews, et al., 2000
chr16:83,959,097-90,294,753	6,335,657	q23.3 - q24.3	Gain	78	83.33333333	0.021	Lockwood WW, et al., 2007; Connie P. Matthews, et al., 2000; Y.W. CHOI, et al., 2007
chr17:7,175,150-8,229,647	1,054,498	p13.1	Gain	59	83.33333333	0.009	N/A
chr17:72,693,870-81,060,000	8,366,131	q25.1 - q25.3	Gain	166	83.33333333	>0.05	Lockwood WW, et al., 2007; Y.W. CHOI, et al., 2007
chr18:18,510,899-43,242,321	24,731,423	q11.1 - q12.3	Loss	70	83.33333333	>0.05	Lockwood WW, et al., 2007; Y.W. CHOI, et al., 2007
chr18:62,450,433-71,009,737	8,559,305	q22.1 - q22.3	Loss	11	83.33333333	>0.05	Lockwood WW, et al., 2007; G Ng, et al., 2007
chr19:1,271,138-4,752,741	3,481,604	p13.3	Gain	105	83.33333333	>0.05	Y.W. CHOI, et al., 2007
chr19:12,747,550-14,740,086	1,992,537	p13.13 - p13.12	Gain	56	83.33333333	>0.05	Gopeshwar Narayan, et al., 2007
chr19:16,170,761-19,780,245	3,609,485	p13.12 - p13.11	Gain	103	83.33333333	>0.05	N/A
chr19:45,216,651-51,316,691	6,100,041	q13.32 - q13.33	Gain	201	83.33333333	>0.05	Lockwood WW, et al., 2007
chr19:55,542,540-56,189,743	647,204	q13.42	Gain	31	83.33333333	>0.05	N/A
chr19:58,530,030-59,114,839	584,810	q13.43	Gain	23	83.33333333	>0.05	N/A
chr20:60,195,293-62,965,520	2,770,228	q13.33	Gain	63	83.33333333	>0.05	Connie P. Matthews, et al., 2000; Y.W. CHOI, et al., 2007; G Ng, et al., 2007
chr21:14,417,523-32,339,619	17,922,097	q11.2 - q22.11	Loss	61	83.33333333	>0.05	N/A
chrX:77,966,491-93,063,726	15,097,236	q21.1 - q21.32	Loss	25	83.33333333	>0.05	Connie P. Matthews, et al., 2000
chrX:120,138,580-127,769,411	7,630,832	q24 - q25	Loss	10	83.33333333	>0.05	Connie P. Matthews, et al., 2000
chrX:152,449,419-153,711,912	1,262,494	q28	Gain	47	83.33333333	0	Gopeshwar Narayan, et al., 2007; Connie P. Matthews, et al., 2000

Eleven of these (5 amplification regions and 6 deletion regions) have not been previously described in cervical cancer (Table 1). A total of 3514 genes were identified, and many tumor related genes, such as *ABL1*, *BCL3*, *CDH1*, *CDKN1C*, *EPHA3*, *ERBB4*, *FOSL1*, *JUNB*, *MLH1*, *MYB*, *p53*, *RB1*, *ROS1*, *SKI*, *TGFBR1* and *THRB*, were located in these 50 chromosomal CNV regions.

### Chromosomal CNVs could contribute to their transcript expression in cervical cancer

**Figure 1:**
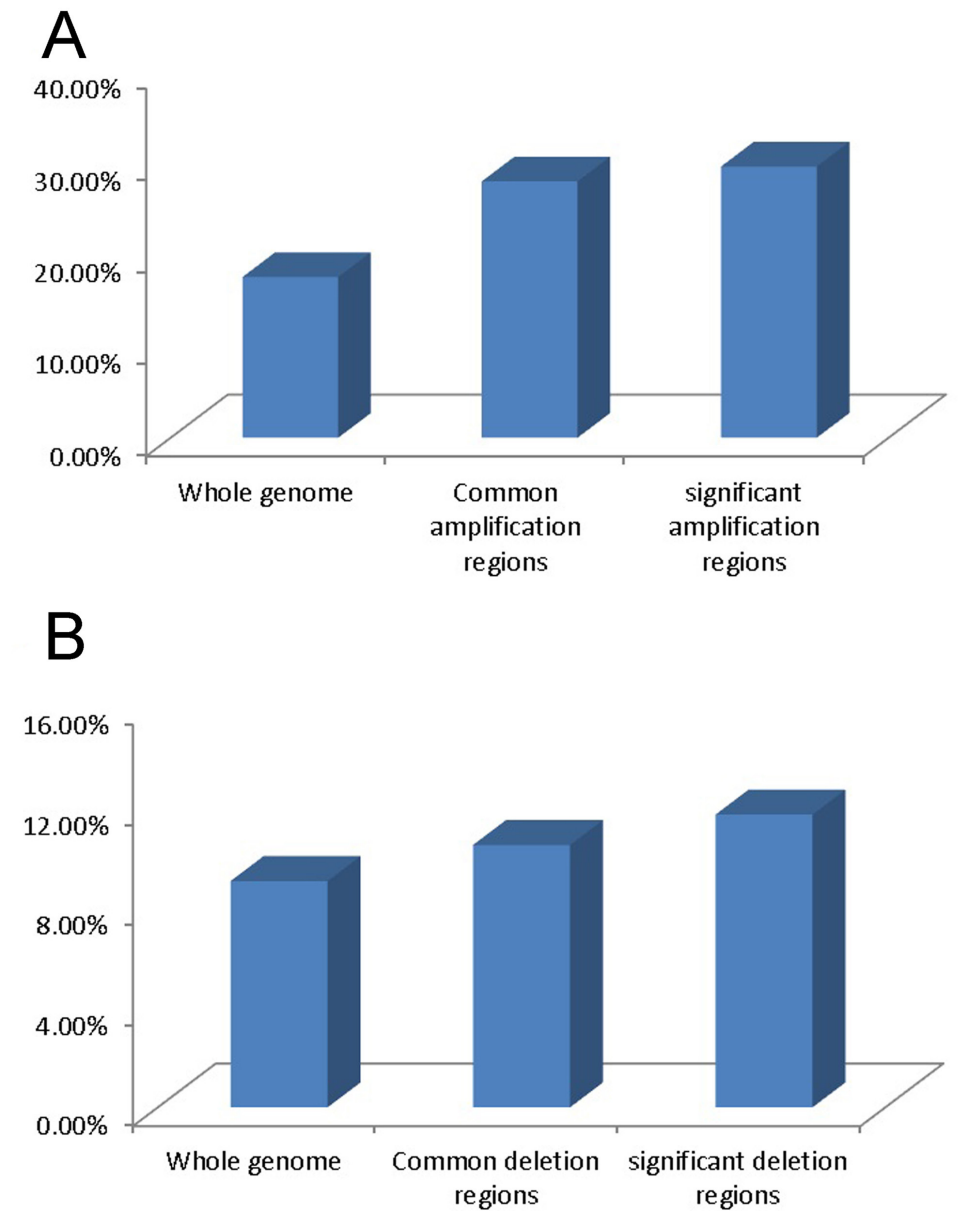
Gene expression variation in different genomic regions **(A)** Percentage of up-regulated transcripts in whole genomic regions, common amplification regions and significant amplification regions. **(B)** Percentage of down-regulated transcripts in whole genomic regions, common deletion regions and significant deletion regions.

To evaluate if there is any association between chromosomal CNVs and gene expression changes in cervical cancer, we analyzed the gene expression profiles of the cervical cancer cell lines and normal cervical epithelium samples. 17.52% of transcripts (7,211 out of 41,152) exhibited a 2-fold over-expression and 9.02% of transcripts (3,712 out of 41,152) displayed a 2-fold down-regulation in 6 cervical cancer cell lines compared with 3 normal cervical epithelium tissues. Within the 21 common genomic amplification regions, 27.94% of the transcripts (772 out of 2,794) showed 2-fold over-expression. In the 13 significant amplification regions, the percentage was 29.56% (459 out of 1,553). In the 29 deletion regions, 10.46% (287 out of 2744) revealed 2-fold down-regulation. In the 2 significant deletion regions, the percentage was 11.67% (7 out of 60) (Figure 1A and 1B). Statistical analysis showed that gene up-regulation or down-regulation was significantly correlated with genomic amplification (P < 0.01, Spearsman correlation test) or deletion (P < 0.01) events. Thus, chromosomal CNVs can contribute to their transcript expression in cervical cancer. Two tumor related genes, *ABL1* and *p53*, which were located in the genomic amplification regions, were found to be over-expressed by at least 2-fold in cervical cancer.

### Profiles differed between cervical cancer cell lines, primary cervical carcinoma and normal cervical epithelium tissues

**Figure 2:**
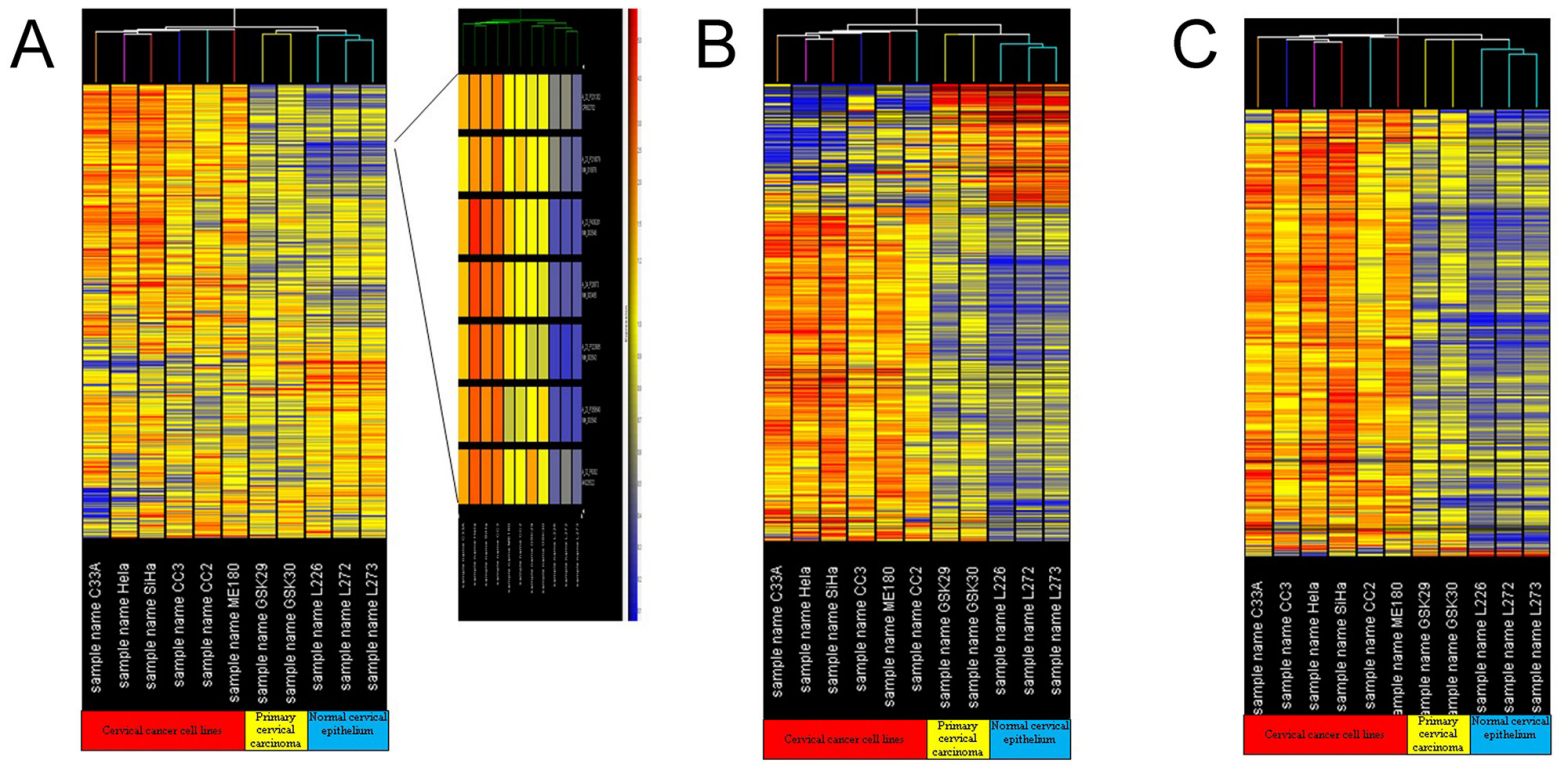
Gene tree clustering analysis **(A)** Gene tree clustering analysis for the gene expression profiles of the cervical cancer cell lines, cervical carcinomas and normal cervical epithelium tissues; **(B)** gene tree clustering analysis for the aberrantly expressed genes in 50 common chromosomal CNV regions in cervical carcinoma; **(C)** gene tree clustering analysis for the aberrantly expressed genes in 15 significant chromosomal CNV regions in cervical carcinoma.

Expression profiles of transcripts across 6 different cervical cancer cell lines and 2 cervical carcinoma tissues and 3 normal, age-matched, cervical epithelium samples were analyzed using a hierarchical clustering algorithm (unsupervised K-means clustering). Cervical cancer cell lines, cervical carcinoma and normal cervical epithelium were divided two main groups (Figure 2A): cervical cancer cell lines for one group, and clinical cervical carcinoma and normal cervical epithelium for the other group. Interestingly, when gene tree clustering analysis was used to analyze genes with aberrant expression within the 50 common chromosome CNV regions and 15 significant chromosome CNV regions, similar results were obtained (Figure 2B and 2C)

### Gene ontology analysis for aberrantly expressed genes

By using a volcano plot in GeneSpring, 9,446 transcripts (27.4%) were found to be changed over two fold in the “Cancer group” (including 6 cervical cancer cell lines and 2 clinical cervical carcinomas) compared with the “Normal group” (including 3 cervical epithelium tissues). Among these genes, 6,001 transcripts were up-regulated by over 2 fold, and 3,445 transcripts were down-regulated by over 2 fold.

### Pathway analysis showed that cell cycle pathways, cell communication pathways and DNA polymerase pathways were significantly affected pathways in cervical cancer

**Figure 3:**
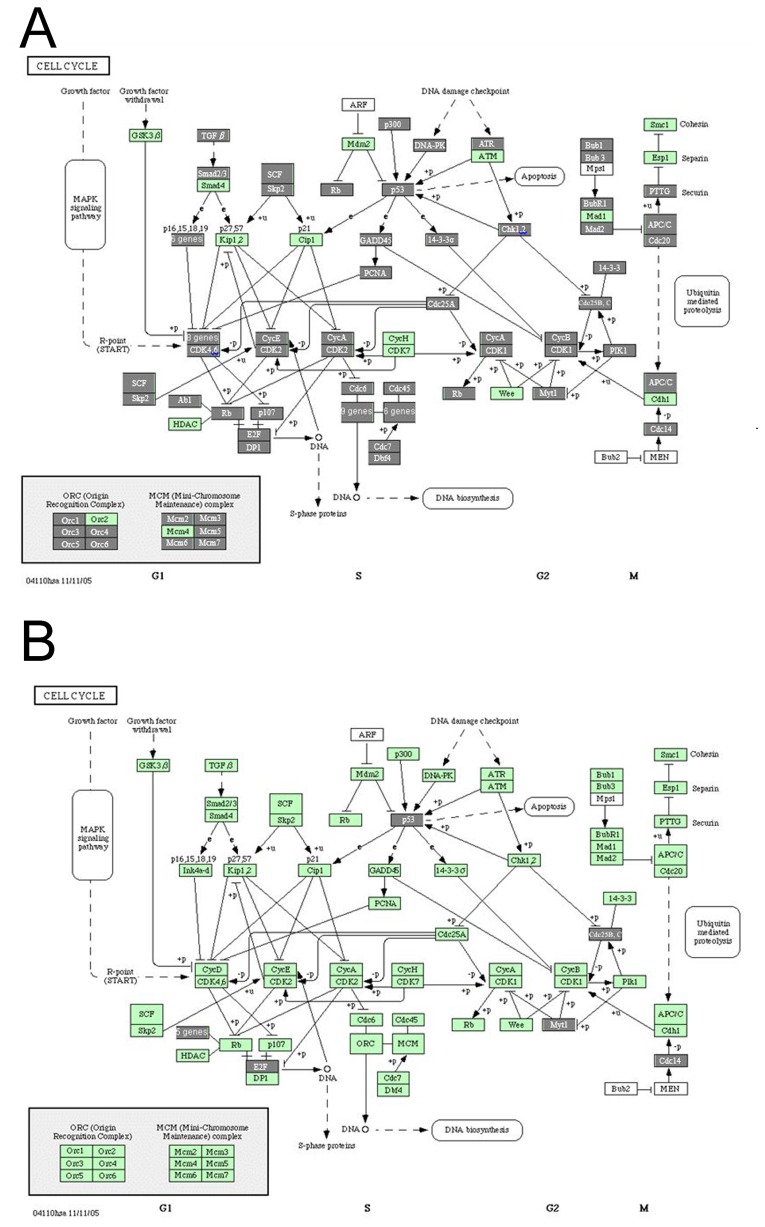
Cell cycle pathway analysis in cervical cancer **(A)** Differently expressed genes involved in the cell cycle pathway in cervical carcinoma; **(B)** differently expressed genes within the significant chromosomal CNV regions involved in the cell cycle pathway in cervical carcinoma. Each rectangle represents one gene. The rectangle covered by gray color indicates that this gene is differently expressed in cervical carcinoma compared with normal cervix.

To further investigate the biological significance of these aberrantly expressed genes, pathway analysis was performed. The analysis results showed that cycle cycle pathways (*P*=1.15e-8), cell communication pathways (*P*=1.15e-8) and DNA polymerase pathways (*P*=1.15e-8) were significantly were affected in the cervical cancer (Table 2). Pathway analysis for the 446 differentially expressed transcripts in the 15 significant chromosomal CNV regions also showed that the cell cycle pathway involved the highest number of transcripts (including *ABL1*, *DUSP9*, *E2F4*, *TP53*, *PKMYT1* and *PPP1CA*) (Figure 3), and the *P* value is 0.00062 (Table 3).

**Table 2:** Pathway analysis for the differentially expressed genes in cervical cancer

Pathway	Number of genes with each pathway	Genelist vs pathway random overlap p-value
Cell cycle - Homo sapiens (human)	111	1.15E-08
Proteasome - Homo sapiens (human)	27	2.66E-08
One carbon pool by folate - Homo sapiens (human)	22	5.11E-07
Cell Communication - Homo sapiens (human)	60	5.85E-07
Pyrimidine metabolism - Homo sapiens (human)	52	6.05E-06
Purine metabolism - Homo sapiens (human)	77	2.27E-05
DNA polymerase - Homo sapiens (human)	19	5.80E-05
Arginine and proline metabolism - Homo sapiens (human)	35	0.000103
Riboflavin metabolism - Homo sapiens (human)	14	0.00036
gamma-Hexachlorocyclohexane degradation - Homo sapiens (human)	17	0.000515
Hematopoietic cell lineage - Homo sapiens (human)	45	0.000654
Valine, leucine and isoleucine biosynthesis - Homo sapiens (human)	10	0.000804
Glycosphingolipid biosynthesis - ganglioseries - Homo sapiens (human)	13	0.000894
Pathogenic Escherichia coli infection - EHEC - Homo sapiens (human)	33	0.000941
Pathogenic Escherichia coli infection - EPEC - Homo sapiens (human)	33	0.000941
Selenoamino acid metabolism - Homo sapiens (human)	23	0.00105
Valine, leucine and isoleucine degradation - Homo sapiens (human)	31	0.00139
Urea cycle and metabolism of amino groups - Homo sapiens (human)	17	0.00172
ECM-receptor interaction - Homo sapiens (human)	46	0.00179
Glycan structures - biosynthesis 2 - Homo sapiens (human)	36	0.00261
2,4-Dichlorobenzoate degradation - Homo sapiens (human)	6	0.00264
Butanoate metabolism - Homo sapiens (human)	24	0.00302
Lysine degradation - Homo sapiens (human)	30	0.00449
Aminoacyl-tRNA biosynthesis - Homo sapiens (human)	17	0.0047
Cell adhesion molecules (CAMs) - Homo sapiens (human)	63	0.0062
Folate biosynthesis - Homo sapiens (human)	22	0.00732
Ascorbate and aldarate metabolism - Homo sapiens (human)	10	0.00742
Histidine metabolism - Homo sapiens (human)	22	0.00918
Alkaloid biosynthesis II - Homo sapiens (human)	14	0.0101
Limonene and pinene degradation - Homo sapiens (human)	18	0.0102
Focal adhesion - Homo sapiens (human)	91	0.0108
Methionine metabolism - Homo sapiens (human)	11	0.0112
Oxidative phosphorylation - Homo sapiens (human)	49	0.012
Citrate cycle (TCA cycle) - Homo sapiens (human)	16	0.0153
Glycosaminoglycan degradation - Homo sapiens (human)	11	0.016
Nitrobenzene degradation - Homo sapiens (human)	10	0.0165
Olfactory transduction - Homo sapiens (human)	17	0.0179
Glycolysis Gluconeogenesis - Homo sapiens (human)	31	0.0197
Ethylbenzene degradation - Homo sapiens (human)	11	0.0221
Ubiquitin mediated proteolysis - Homo sapiens (human)	26	0.0236
Glyoxylate and dicarboxylate metabolism - Homo sapiens (human)	9	0.0243
Arachidonic acid metabolism - Homo sapiens (human)	25	0.0306
N-Glycan biosynthesis - Homo sapiens (human)	21	0.033
Chondroitin sulfate biosynthesis - Homo sapiens (human)	7	0.037
Linoleic acid metabolism - Homo sapiens (human)	17	0.0415
Pentose phosphate pathway - Homo sapiens (human)	14	0.0465
Apoptosis - Homo sapiens (human)	38	0.0467
Protein export - Homo sapiens (human)	8	0.0498

**Table 3:** Pathway analysis for the differentially expressed genes within the 15 significant chromosome CNV regions in cervical cancer

Pathway	Number of common genes with each pathway	Genelist vs pathway random overlap p-value
Cell cycle - Homo sapiens (human)	11	0.000622
Purine metabolism - Homo sapiens (human)	7	0.0126
Axon guidance - Homo sapiens (human)	6	0.0365
Insulin signaling pathway - Homo sapiens (human)	6	0.0386
Selenoamino acid metabolism - Homo sapiens (human)	5	0.000365
Tyrosine metabolism - Homo sapiens (human)	5	0.00382
Glycerophospholipid metabolism - Homo sapiens (human)	5	0.00419
Tryptophan metabolism - Homo sapiens (human)	5	0.0137
Nitrobenzene degradation - Homo sapiens (human)	4	0.000101
Aminophosphonate metabolism - Homo sapiens (human)	4	0.000199
Histidine metabolism - Homo sapiens (human)	4	0.00452
Androgen and estrogen metabolism - Homo sapiens (human)	4	0.00747
Glycan structures - biosynthesis 2 - Homo sapiens (human)	4	0.0281
Sulfur metabolism - Homo sapiens (human)	3	0.000724
Ethylbenzene degradation - Homo sapiens (human)	3	0.00309
RNA polymerase - Homo sapiens (human)	3	0.00781
1- and 2-Methylnaphthalene degradation - Homo sapiens (human)	3	0.0136
Benzoate degradation via CoA ligation - Homo sapiens (human)	3	0.0153
Limonene and pinene degradation - Homo sapiens (human)	3	0.0153
Pyruvate metabolism - Homo sapiens (human)	3	0.0362
Bisphenol A degradation - Homo sapiens (human)	2	0.0233
Glycosphingolipid biosynthesis - ganglioseries - Homo sapiens (human)	2	0.03
Dentatorubropallidoluysian atrophy (DRPLA) - Homo sapiens (human)	2	0.0323
Parkinson's disease - Homo sapiens (human)	2	0.0323

## DISCUSSION

In our genome-wide CNV analysis, we identified 50 frequently altered genomic regions (ranging from 0.5 Mb to 80 Mb), of which 11 have not been previously described in cervical cancer (Table 1). These differences in our results may be due to the different platforms of assay, different settings of analysis or the different cervical cancer cells. In these 50 commonly altered genomic regions, 3514 genes are included. Some of these, especially oncogenic or tumor suppressor genes, may be associated with the development of cervical cancer.

The gene tree clustering result suggested that during the development of cervical carcinoma, gene expression significantly changes, and cervical carcinoma can be distinguished from normal cervical epithelium tissue by clustering analysis of the gene expression profile. Since the cervical cancer cell lines were separate from primary cervical carcinoma and normal cervical epithelium tissue, we assumed that extended culturing of cervical cancer cell lines may also significantly alter their gene expression profiles.

The integrated analysis of genome-wide chromosomal copy number changes and gene expression profiling indicated that the identified CNVs could contribute to the expression of some but not all genes (Figure 1). This finding is consistent with a report by Vazquez-Mena et al. which used a different array platform to detect the correlation between CNVs and gene expression variation in cervical cancer cell lines. Other factors, such as epigenetic changes or transcription factors, may also contribute to variation of gene expression in cervical cancer [4]. Pathway analysis indicated that significant changes of some pathways, especially those involving the cell cycle, may be involved in the pathogenesis of cervical cancer.

*ABL1 and p53*, the two cell cycle pathway tumor-related genes which were located in the significant genomic amplification regions, were found to be overexpressed at least 2-fold in cervical cancer. *ABL1,* which was located in the chr9:128,223,213-139,309,447 genomic amplification region, plays a role in apoptosis. *p53* is a well-known tumor suppressor gene, and an increase in *p53* levels plays a critical role in the induction of genes that results in cell cycle arrest [7], allowing repair of damaged DNA or activation of apoptotic pathways [8]. In cervical cancer with high risk of HPV-infection, the E6 protein from high-risk HPV can bind to tumor suppressor protein p53 for rapid degradation via a cellular ubiquitin ligase [9]. Other studies have indicated that p53 protein over-expression is not common or associated with survival in cervical carcinoma [10, 11]. However, from our array CGH and gene expression array data, *p53* was located in the chr17:7,175,150-8,229,647 genomic amplification region and was over-expressed at the mRNA level (the median of the expression of *p53* in cervical cancer cell lines was 1.316 [from 0.82 to 3.38); the median of the expression of *p53* in normal cervical epithelium was 0.295 [from 0.153 to 0.485]). This suggests that gene dosage of *p53* contributes to RNA over-expression in some cervical cancers.

Our results demonstrated that over-expression of transforming growth factor-beta 1 (TGF-β1), a gene important in cell cycle pathways, may be due to a chromosomal CNV. TGF-β1 was amplified and was also over-expressed (>2 fold) in the cancer group compared with the normal group. TGF-β1 is involved in many different critical processes, such as embryonic development, cellular maturation and differentiation, wound healing, immune regulation and inflammation. TGF-β1 is a potent inhibitor of cell proliferation at the beginning of carcinogenesis [12, 13]. When cells become resistant to TGF-β1, tumor growth may be enhanced and metastasis promoted via immune evasion and angiogenesis. An increased expression of TGF-β1 has been found in cervical cancer. Kirma et al. suggested that TGF-β1 may be a factor in inducing over-expression of an oncogene, c-fms. Blocking c-fms has been demonstrated to result in increased apoptosis and decreased motility in cervical cancer [14].

Matrix metalloproteinases (MMPs) play an important role in the enhancement of tumor-induced angiogenesis. Our aCGH data showed that 9 MMP genes (MMP1, 3, 7, 8, 10, 12, 13, 20 and 27) located within 11q22 are amplified in Caski and SiHa cell lines, consistent with Lockwood’s findings. Further analysis revealed that MMP14, 23B and 25 were located in significant genomic amplification regions and MMP1, 15, 17 and TIMP1 in all our 6 cervical cancer cell lines. This is consistent with previous work reporting over-expression of MMP12 and MMP15 [15-19].

In summary, we have identified several chromosomal CNV regions and demonstrated that chromosomal CNVs are a common phenomenon which can affect the level of RNA expression in cervical cancer. Pathway analysis for the aberrantly expressed genes suggested that significant changes of some pathways, especially those involving the cell cycle, may contribute to the pathogenesis of cervical cancer. This study provided some clinical significance for us to have a better understanding of cervical cancer pathogenesis.

## MATERIALS AND METHODS

### Cervical cancer cell lines and specimens

Six human cervical cancer cell lines (HeLa, SiHa, C33A, ME180, CC2 and CC3) were used for aCGH and gene expression array. Five clinical specimens, including three normal cervical tissues and two cervical carcinoma specimens (FIGO stage: II_A_), were collected from patients (aged between 36-42 years old) at the Department of Obstetrics and Gynaecology at the Prince of Wales Hospital in Hong Kong from January 2014 to December 2014. Informed consent was obtained from all participating subjects, and Institutional Review Board approval was obtained.

### Tissue micro-dissection

Micro-dissection was used as described in our previous study [5]. Briefly, the tissue specimens were frozen in OCT cryomoulds (SAKURA, Japan), sectioned (8 μm) by a cryostat at -20°C (Leica Corp., CRYOCUT 1800), and then mounted onto glass slides (SAIL BRAND, Cat No 7105) at room temperature. Sections were stained by 0.1% methyl green (Sigma) and micro-dissected using a sterile surgical blade (AESCULAP) and collected immediately for further experiments.

### Microarray comparative genomic hybridization analysis

Microarray comparative genomic hybridization using Human Genome CGH Microarray Kit (4x44K) (Agilent Technologies, Santa Clara, CA, USA) was used for identifying chromosomal CNV of 6 cervical cancer cell lines and 3 normal cervical samples as per the manufacturer’s protocol. Briefly, genomic DNA of cervical cancer cell lines was extracted using a DNeasy Blood & Tissue Kit (QIAGEN, Cat No. 69506). 1 μg genomic DNA of test sample and 1 μg human sex-matched control DNA as a reference sample (Promega G1521A; Lot no. 20929604) were digested using Alu I and Rsa I. This was followed by fluorescent labeling, clean-up of labeled genomic DNA, microarray hybridization and scanning. The data were extracted using the Agilent Feature Extraction (FE) v11.0 program. After calculating the background signal, non-uniform signal and the average raw signal on each probe, the resulting data files were generated and transferred to the bioinformatics software, Nexus Copy Number version 6.1 (BioDiscovery, Inc., El Segundo, CA, USA) for analysis [6].

### Gene expression analysis

The Whole Human Genome Microarray Kit, 4x44K (G4112F) was used to probe gene expression in 6 cervical cancer cell lines, 2 cervical carcinomas and 3 normal cervical epithelium tissue samples. The resulting data files were generated and transferred to GeneSpring GX version 11.5 (Agilent Technologies, Santa Clara, CA, USA) for further analysis.

### Reverse transcription PCR (RT-RCR)

RT-RCR was peformed for the identified gain and loss genes both on expression level and on genome level, normalized by GAPDH and B-globin (Forward: 5’-GAAGAGCCAAGGACAGGTAC-3’, Reverse: 5’-CAACTTCATCCACGTTCACC-3’), B2M (Forward:5’-TGCTGTCTCCATGTTTGATGTATCT-3’;Reverse:5’-TCTCTGCTCCCCACCTCTAAGT-3’), respectively.

### Statistical analysis

For the Microarray Comparative Genomic Hybridization Analysis, 0.37 was used as the cut off value for amplification or 0.5 for deletion of a single probe. Putative chromosome copy number changes were defined by intervals of three or more adjacent probes with log_2_ ratios suggestive of a deletion or duplication when compared with the log_2_ ratios of adjacent probes. The p value for significant difference was set to less than 0.05 to reduce the false discovery rate (FDR). For **Gene Expression Analysis,** the cut-off value defining an aberrant change of gene expression was set at 2 fold for data analysis. Pathway analysis for the gene expression data was performed by GeneSpring GX version 11.5, and the pathway was downloaded from the KEGG database (ftp://ftp.genome.jp/pub/kegg/).

## Abbreviations

CNV: Copy number variations
HPV: Human papillomavirus
CGH: Comparative genomic hybridization
TGF-β1: Transforming growth factor-beta 1
MMPs: Matrix metalloproteinases
FDR: False discovery rate
